# Adhesion Studies during Generative Hybridization of Textile-Reinforced Thermoplastic Composites via Additive Manufacturing

**DOI:** 10.3390/ma14143888

**Published:** 2021-07-12

**Authors:** Johanna Maier, Christian Vogel, Tobias Lebelt, Vinzenz Geske, Thomas Behnisch, Niels Modler, Maik Gude

**Affiliations:** Institute of Lightweight Engineering and Polymer Technology, University of Dresden, Holbeinstraße 3, 01307 Dresden, Germany; Christian.Vogel@tu-dresden.de (C.V.); Tobias.Lebelt@tu-dresden.de (T.L.); Vinzenz.Geske@tu-dresden.de (V.G.); Thomas.Behnisch@tu-dresden.de (T.B.); Niels.Modler@tu-dresden.de (N.M.); Maik.Gude@tu-dresden.de (M.G.)

**Keywords:** additive manufacturing, generative hybridization, multi-material design, fused layer modeling, functionalization, thermoplastic composite, adhesion

## Abstract

Generative hybridization enables the efficient production of lightweight structures by combining classic manufacturing processes with additive manufacturing technologies. This type of functionalization process allows components with high geometric complexity and high mechanical properties to be produced efficiently in small series without the need for additional molds. In this study, hybrid specimens were generated by additively depositing PA6 (polyamide 6) via fused layer modeling (FLM) onto continuous woven fiber GF/PA6 (glass fiber/polyamide 6) flat preforms. Specifically, the effects of surface pre-treatment and process-induced surface interactions were investigated using optical microscopy for contact angle measurements as well as laser profilometry and thermal analytics. The bonding characteristic at the interface was evaluated via quasi-static tensile pull-off tests. Results indicate that both the bond strength and corresponding failure type vary with pre-treatment settings and process parameters during generative hybridization. It is shown that both the base substrate temperature and the FLM nozzle distance have a significant influence on the adhesive tensile strength. In particular, it can be seen that surface activation by plasma can significantly improve the specific adhesion in generative hybridization.

## 1. Introduction

Multi-material structures are a target-oriented approach to produce highly lightweight structures, especially for the aviation and automotive sector. Such multi-material structures enable the integration of functional elements and the improvement of mechanical properties and allow new design freedom [1]. In particular, modern car body structures increasingly use intrinsic hybrid structures and combination processes, in which flat semi-finished products made of metal- or textile-reinforced thermoplastic are formed and functionalized using injection molding technology [2,3,4]. This requires tooling systems that are very cost intensive and unsuitable for efficient production in the low volume range with a high number of variants.

However, in order to be able to implement flexible manufacturing processes while at the same time achieving high resource efficiency, the use of additive manufacturing technologies is an ideal solution. This allows the semi-finished products to be functionalized after shaping without additional tools in a generative hybridization process, as shown by [5,6] using fused layer modeling (FLM) and [7] using liquid resin print as well as [8,9,10,11] directly printing onto textile substrates via FLM. An essential part of the generative hybridization process of such multi-material structures is the joining of the various semi-finished products. Therefore, pre-treatment strategies are necessary to ensure a highly loadable connection of the hybrid structure.

Due to their low surface energy, thermoplastics in particular have limited adhesion capabilities [12]. The required surface energy is determined by dispersal and polar fractions, with the polar fraction in particular being decisive for the adhesion strength. By means of a suitable surface pre-treatment, adhesion can be improved by adjusting the polar fractions of both joining partners [12]. Established physical pre-treatment methods are surface coating [13,14,15], flame treatment [16], and plasma treatment [17,18,19], as well as corona and UV/ozone [20] pre-treatment. Based on the research results regarding the hybridization of textile-reinforced thermoplastic components using injection molding technology [21,22,23], the focus is on the influence of pre-heating and plasma activation of the base substrate. The authors of [2] demonstrated that atmospheric plasma treatment in particular is highly suitable to achieve high bond strength between glass fiber reinforced polyamide 6 composite (PA6 GF) sheets hybridized with unreinforced polyamide 6 (PA6).

In order to fully exploit the lightweight potential and achieve maximum bond strengths during generative hybridization, a more detailed consideration of the interfaces between the individual components is necessary. This requires an in-depth understanding of the process control as well as the investigation of different pre-treatment strategies in order to evaluate and optimize the bonding properties between the materials and to achieve a load-bearing composite with maximum bond strengths.

## 2. Materials and Methods

### 2.1. Materials

Specimen samples were manufactured using a PA6 filament (Nylon Black) produced by Ultimaker (Utrecht, The Netherlands). The molecular structure of PA6 results in a high hygroscopy compared to that of other thermoplastic materials. This absorbed water can lead to a change in material properties or the sample’s geometry as well as a degassing of the filament during the printing process. Consequently, the printing quality is reduced due to the formation of air bubbles. To prevent this, the material was dried in an oven for 12 h at 80 °C. A textile-reinforced thermoplastic composite (TPC) sheet with the designation “FG 217” was used as a base substrate. It consists of PA6 and 47 vol % glass fiber (GF) woven in a (2/2) twill (Tepex Dynalite 102RG600 by Bondlaminates (Brilon, Germany), 47 vol % glass fiber content, 2 mm thickness).

### 2.2. Specimen Preparation

The bonding strength of generatively manufactured hybrid PA6 GF specimen was characterized as dependent on different surface treatments of the substrate (TPC sheet). Building on results of earlier research concerning the hybridization of textile-reinforced thermoplastic components [3,4,21,22,23], the pre-heating and plasma activation of the TPC sheet were chosen as the focus of this work. Furthermore, the influence of the distance between the FLM nozzle and the TPC surface on the bonding strength was investigated, as it was seen to significantly affect the adhesion behavior of textile substrates during hybridization [17].

#### 2.2.1. Nozzle Distance

**Figure 1:**
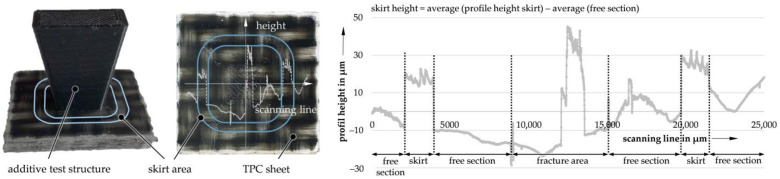
Profilometer measurement and calculation of FLM nozzle distance via skirt height.

In the functionalization of hybrid structures using FLM processes, the quality of the applied first layer in particular plays an essential role in the formation of an optimal bond [5]. During the generative hybridization of TPC, this was purposefully influenced by the distance between the nozzle and the surface of the TPC. The distance of the FLM nozzle was determined at the so-called skirt. This structure is printed around the actual object without direct contact with the aim to ensure a continuous material flow right at the start of the print. The skirt is exactly one layer high, which allows a correlation of FLM nozzle distance and bonding strength; e.g., with a layer height of 0.2 mm and a measured skirt height of 0.1 mm, the FLM nozzle distance is −0.1 mm. The analysis and calculation of the skirt height are shown in Figure 1.

The height of the skirt was measured using a laser profilometer µScan manufactured by Nanofocus (Oberhausen, Germany). This method is a non-destructive and non-invasive means of characterizing the topology and structure of surfaces. Pre-adjustment of the skirt height was tuned in 0.05 mm steps using feeler gauges. A detailed assignment was achieved by the following profilometry since an exact presetting could not be made due to minimal manufacturing differences of the TPC sheets.

#### 2.2.2. Print Bed Temperature

To ensure an even temperature distribution, substrates were heated using an aluminum heating plate (100 W, max. temperature of 250 °C). The print bed was pre-heated to temperatures of 100 °C, 120 °C, 140 °C, and 160 °C. For the duration of the print process, the temperature was kept stable at the set value. To achieve the desired temperature on the TPC surface, a temperature sensor (type K) was applied to the surface throughout the printing process. Depending on the ambient process conditions, the heating process varied in duration. Additionally, the TPC sheet material was dried beforehand for 12 h at 80 °C.

#### 2.2.3. Plasma Treatments

To evaluate the bonding behavior of generatively hybridized structures on a TPC sheet, the latter was cleaned using isopropyl alcohol and pre-treated and activated via plasma treatment. The process was conducted using a cold atmospheric-pressure plasma machine from Plasmatreat GmbH (Steinhagen, DE, Germany). The plasma nozzle is moved across the surface of the specimen via a robotic system during the treatment. At the same time, the head holding the plasma nozzle is rotated at a predefined rotational speed. Due to the plasma nozzle geometry, the influenced area on the specimen surface exhibits a circular shape as evaluated for optimal bonding strength in [2].

To validate the effect of the surface treatment, the bonding strength between the TPC sheet and the FLM-deposited structures was determined. Using a 2-level fractional factorial design (FFD) approach, interaction effects between the process parameters were analyzed [2]. To that end, minimal and maximal values in a recommended range were used as stages for each factor. The modified process parameters and their ranges are shown in Table 1.

Due to recommendations given by Plasmatreat GmbH, the number of examined parameters could be reduced from eight to six, as fixed values were used for the plasma cycle time (PCT; relative ignition impulse length) and the frequency. Since the smallest possible treatment width is about 25 mm at a plasma nozzle head distance of 1 mm and the sample size is only 10 × 10 mm, the pitch (overlap of parallel paths during plasma treatment) can also be disregarded. Hence, the number of process factors was further reduced to five.

**Figure 2:**
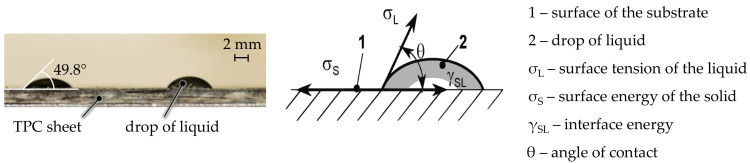
Applied drop for contact angle measurement (**left**) and analyzed properties (**right**) [24] (copyright accessed from Berlin Beuth Verlag GmbH).

To assess the influence of the plasma treatment on the surface, contact angle measurements were performed (Figure 2). For this purpose, 5 separate drops of water were applied to each sample directly after the plasma treatment, and the average contact angle was measured. Due to the increase in the surface energy of the material, an improved specific adhesion could be achieved (low contact angle).

The resulting configurations of different samples with varying process parameters of plasma treatment A–E (cf. Table 1) of the 2-level FFD are shown in Table 2.

**Figure 3:**
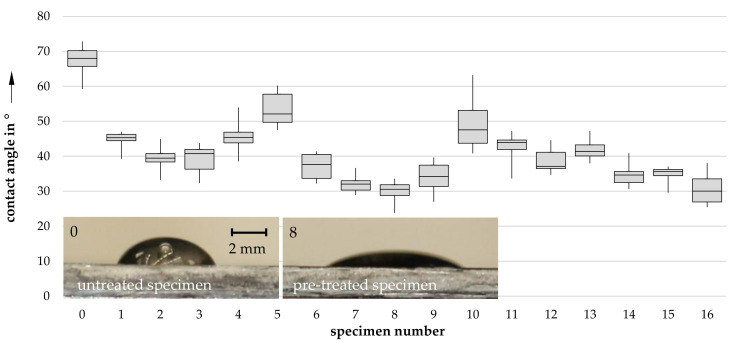
The resulting 2-level fractional factorial contact angle measurements for specimen with different plasma treatment (sample designations as seen in Table 2).

The resulting contact angles are plotted in Figure 3. The sample designation “0” identifies a non-treated sample, showing a significantly higher contact angle compared to that of plasma-treated samples (1 through 16).

Interpretation of the 2-level fractional factorial design’s result was conducted using the so-called contrast method [25]. The contrast is a degree of effect, meaning it describes the influence of one factor on the target parameter. This dimensionless quantity is calculated by subtracting the mean value of the first level of one parameter from the mean value of the second level of that same parameter. The resulting contrast and effect values are shown in Table 3.

Table 3 is also used to calculate the interaction of parameters with one another. To avoid unnecessary complexity, only two parameters are connected at a time. The degree of interaction is also given by the contrast. Once again, two mean values are used for its calculation. Plasma nozzle speed and plasma nozzle distance (contrast 2.3) are shown to have a high dependency on each other, with the positive algebraic sign of the interaction effect coinciding with an amplification. On the other hand, the effect of volumetric flow of the ionization gas and voltage was calculated to a value of −3.9. This means that even though the voltage affects the contact angle somewhat (0.5), the interaction effect of the combination is completely negated by the voltage (−3.2). The same behavior can be observed for all other parameter interactions as well. Thus, the plasma nozzle distance and the plasma nozzle head speed exhibit the highest influence on the contact angle.

### 2.3. Development of a Hybrid Testing Structure

**Figure 4:**
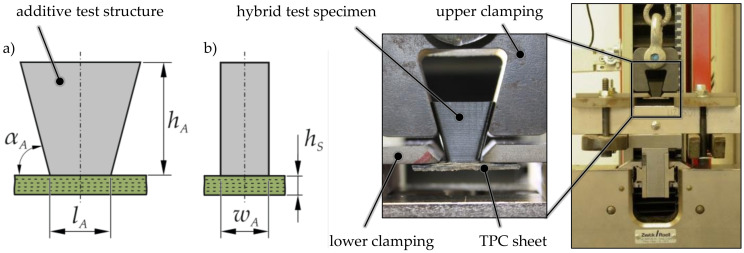
Adapted sample geometry: (**a**) front view (h_A_ = 17.5 mm; l_A_ = 10 mm; α_A_ = 75°); (**b**) side view (h_S_ = 2 mm; w_A_ = 10 mm) and test setup.

Process-optimized testing strategies and sample geometries are necessary to determine and assess realistic bonding strengths of generative hybridization. Similar to fiber-reinforced polymers, parts manufactured using the FLM process exhibit strong anisotropies due to their layer-based structure. For example, a tensile load perpendicular to the layer plane can result in delamination inside the sample, prohibiting a qualitative evaluation of the joining zone’s bonding strength. Based on the proven tensile test for metal–polymer composites [26], an adapted testing procedure, as well as a suitable equipment, has been developed (Figure 4). To avoid delamination inside the sample, a trapezoid test geometry was used. This guarantees load application via positive mechanical engagement and realizes the highest stress in the joining zone since it exhibits the smallest cross-section. The tests were performed using a universal testing machine Zwick Roell 2.5 kN (Zwick GmbH, Ulm, Germany) with a strain rate of 1 mm/min. A bending-free clamping of the specimen was guaranteed using a chain as a flexible load transmission.

The test structures were printed with a brass nozzle and a dried PA6 filament (Nylon Black by Ultimaker (Utrecht, The Netherlands). Slicing of the sample geometry’s STL file was performed using the manufacturer’s slicing software Cura (version 4.4) (Table 4).

## 3. Results

### 3.1. Adhesive Tensile Strength

#### 3.1.1. Influence of FLM Nozzle Distance

**Figure 5:**
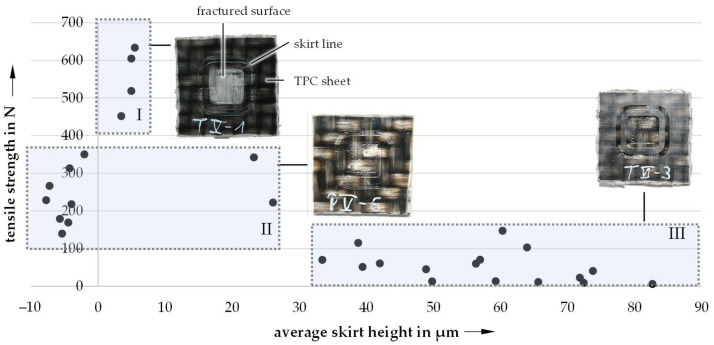
Influence of the FLM nozzle distance on the bonding strength by means of averaged skirt heights.

During the experiments, it was shown that penetration of the FLM nozzle into the surface of the substrate could be advantageous for the bonding strength between the TPC sheet and the printed PA6 structure. The FLM nozzle temperature when melting the polymer filament using an FLM printer can be sufficiently high to allow the FLM nozzle to drive to negative distances, melting the surface in the process. The penetration depth of the FLM nozzle height is equivalent to the height of the printed skirt (and the FLM nozzle height). The bonding strengths as a function of the skirt height are depicted in Figure 5.

As a result of the experiments shown in Figure 5, three distinct regions were identified. Region I results in partially melted surfaces without material removal (SMP—surface partially melted). Samples subjected to these parameters exhibit the highest bonding strengths with values ranging between 4.5 to 6.5 MPa. Region II (MA—matrix abrasion) shows bonding strengths from 1.5 to 3.5 MPa. Due to the FLM nozzle distance being too low, the matrix material of the TPC sheet was stripped in the process. However, the resulting bonding strengths of region III (ME—material extrusion) are only between 0 and 1.5 MPa. This is caused by high nozzle distances leading to poor adhesion. To ensure consistent reproducibility, the following tests were therefore carried out with two settings for the FLM nozzle distance. The SMP samples were prepared with −0.1 mm and the ME samples with +0.1 mm FLM nozzle distance.

#### 3.1.2. Influence of Print Bed Temperature

**Figure 6:**
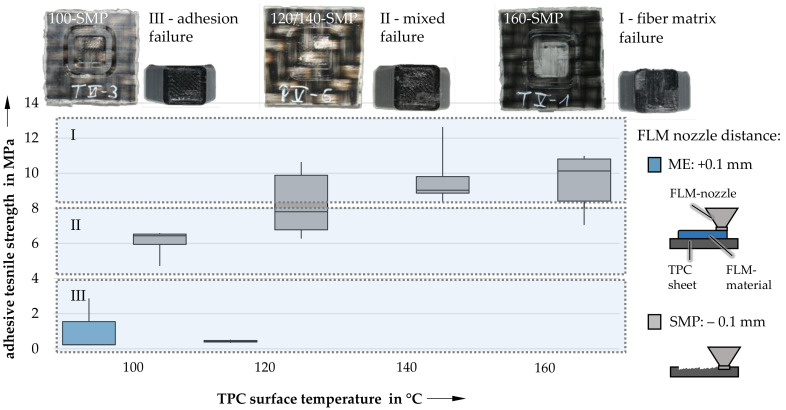
Bonding strengths as a function of TPC surface temperature.

Along the lines of FLM nozzle distance, surface temperatures show an equally high influence on the bonding strengths of the hybrid composite (Figure 6). Increasing the temperature leads to a significant increase in bonding strength up to a temperature of 140 °C. This observation is accompanied by a shift in the mode of failure: samples produced with a surface temperature of 100 °C exhibit almost exclusively adhesion failure, while tests of samples with a surface temperature of 120 °C result primarily in a mixed-mode failure. Samples created at the highest temperatures of 140 and 160 °C even show a fiber–matrix bonding failure, suggesting a significant increase in bonding strength in the joining zone. This can be further enhanced by optimizing the distance of the FLM nozzle to the TPC surface. Given the FLM nozzle distance of 0.1 mm as typically established in the FLM process (ME samples), the surface of the TPC sheet is only partially melted as a result of the heat of the extruded material. On the other hand, direct contact of the nozzle with the surface (SMP samples) leads to an increased input of heat into the TPC sheet, as both the FLM nozzle and the extruded material contribute to the melting of the surface. The increased diffusion of polymer chains can lead to structural changes in the joining zone, resulting in enhanced specific adhesion [21]. It is, however, notable that samples with a surface temperature of 160 °C exhibit slightly diminished bonding strengths compared to those of 140 °C samples.

#### 3.1.3. Influence of Plasma Treatment

Incorporating the results of already conducted experiments, a set of parameter combinations was selected for the plasma treatment (PT) of samples (Table 5). To validate the plasma treatment’s influence, a wide range of altered surface energies was used.

**Figure 7:**
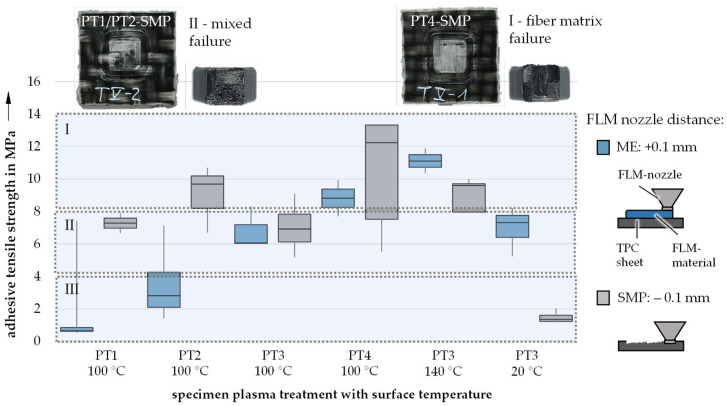
Influence of plasma treatment on bonding strengths.

In addition to the impact of surface temperature and FLM nozzle distance on the bonding strength, plasma treatment of the surface plays a major role in controlling the connection between the TPC and the FLM-deposited PA6. As shown in Figure 7, activating the surface using plasma has the potential to significantly increase the specific adhesion of the generative hybridization. Raising the surface energy (with PT1 exhibiting the lowest and PT4 the highest wettability) improves the bonding behavior on a molecular level and allows for a better connection between the TPC sheet and the FLM-generated structure. For example, it was possible to increase the bonding strength by a factor of 4 while keeping the surface temperature (100 °C) and the FLM nozzle distance (0.1 mm; ME sample) constant simply by choosing optimal parameters for the plasma treatment (comparing PT1 and PT4 in Figure 7). Samples whose pre-heated surfaces were submitted to direct contact with the FLM nozzle (SMP samples) also showed an increase in bonding strength. Furthermore, bonding strengths were significantly improved by plasma-treated surfaces with lower temperatures at FLM nozzle distances of 0.1 mm. This is especially interesting in regard to enabling generative hybridization with conventional FLM manufacturing strategies.

### 3.2. Interface Analysis

**Figure 8:**
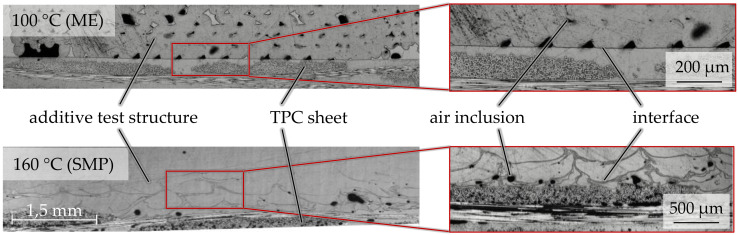
Samples of test specimen printed at 100 (**top**) and 160 °C (**bottom**).

The samples for interface analysis were prepared and investigated using optical microscopy to further analyze the influence of the pre-treatment and process parameters. As an example, Figure 8 illustrates the different structure and interface formation of two samples with a surface temperature of 100 °C (ME) and 160 °C (SMP).

The sample with a surface temperature of 100 °C exhibits a clearly defined line between the TPC sheet and the FLM-generated test specimen. Furthermore, a chain of pores is visible along the boundary in the printed material resulting in a reduced joining area. On the other hand, the surface of the TPC sheet remains almost unchanged.

The sample with a surface temperature of 160 °C exhibits a melted TPC matrix in the surface area resulting in a tooth-like pattern due to the high temperature. Consequently, no clear boundary is visible between the matrix material and the printed test specimen. At the same time, a lot of matrix material is displaced by extruded material, resulting in the interconnection being defined more by the adhesion of the printed material to the substrate’s fibers than to the matrix. This matches the observation of the 160 °C samples showing lower bonding strengths when compared to those printed at a surface temperature of 140 °C, as the latter exhibit even less porosity along the interface. Furthermore, the junction zones of 140 °C samples are significantly more homogenous than those of all other examined temperatures.

## 4. Discussion

The test results show that both the pre-heating of the TPC sheet and the distance of the FLM nozzle have a clear influence on the achievable bond strengths. Thus, it can be seen that with increasing temperature of the TPC surface up to 140 °C, the adhesive tensile strength can be significantly increased. This is also reflected in the failure modes shown. The specimen with a TPC sheet pre-heated to 100 °C fails almost completely with pure adhesion failure. In contrast, in specimens with a surface temperature of 120 °C, a mixed-mode failure occurs, and at 140 °C and 160 °C, even a fiber–matrix bonding failure occurs, which is indicated by much better adhesion in the joining zone. This is further influenced by the distance between the FLM nozzle and the TPC sheet. With an FLM nozzle distance of 0.1 mm to the surface established in the FLM process, the surface is only slightly melted by the material application. In contrast, direct contact of the FLM nozzle with the surface results in melting by both the TPC surface and the discharged material. The resulting structural changes in the joint area can lead to increased diffusion of the polymer chains, which increases the specific adhesion to some extent [27].

In addition to the proven influence of the TPC surface temperature and FLM nozzle distance on the adhesive tensile strength, a significant influence of the bonding properties by plasma pre-treatment of the TPC surface can also be seen. It becomes clear that surface activation by means of plasma can enormously improve the specific adhesion during generative hybridization. The increased surface energy improves the bonding behavior at the molecular level and enables a firm bond between the TPC sheet and the FLM structure. Thus, at a constant surface temperature of 100 °C and an FLM nozzle distance of 0.1 mm, an increase in the adhesive tensile strength by a factor of four can be achieved with the corresponding plasma configuration. In the case of direct contact between the FLM nozzle and the pre-heated TPC surface, a positive influence on the adhesive tensile strength can also be seen. In addition, plasma pre-treatment of the cold surface enables a significant increase in adhesive tensile strengths at an FLM nozzle distance of 0.1 mm and is thus particularly suitable for generative hybridization processes with classic FLM manufacturing strategies.

In order to reach an optimized process with high bonding properties of the hybrid structure, a combination of plasma treatment with a surface temperature of 140 °C and a nozzle distance of 0.1 mm has proven to be most reproducible and effective. Since plasma pre-treatment is a time- and cost-extensive process and surface temperatures of 140 °C are not always efficiently reachable, good results with nearly high adhesive tensile strengths can be achieved without plasma pre-treatment and by a reduced surface temperature of 100 °C in combination with the FLM nozzle distance of −0.1 mm. Since reproducibility is currently low due to the more complex setup of the FLM nozzle distance, this can be improved by an automated distance control and seems more efficient compared to the more expensive plasma pre-treatment, especially in the targeted low-volume range.

## 5. Conclusions

The generative hybridization process presented for textile-reinforced thermoplastic components demonstrates the high potential of generative processes for resource saving and, at the same time, highly flexible production of future multi-material lightweight structures. In addition, the use of appropriate pre-treatment measures offers opportunities to selectively adjust the bond strengths within the generatively hybridized component. Based on the developments presented, generative hybridization processes are to be further enabled for economic and flexible use in complex lightweight construction applications.

## Figures and Tables

**Table 1:** Process parameters of plasma treatment and factors of fractional factorial design.

Parameter	Range	Level 1	Level 2
A: translational plasma nozzle speed	4–16 m/min	4	16
B: distance from plasma nozzle to the surface	4–14 mm	4	14
pitch	22–24 mm		
C: volumetric flow of the ionization gas	30–60 L/min	30	60
D: plasma generation voltage	280–300 v	280	300
frequency	21 kHz		
plasma cycle time (PCT)	100%		
E: rotational speed	1000–2600 U/min	1000	2600

**Table 2:** Sample configuration with varying process parameters of plasma treatment A–E (cf. Table 1) and resulting contact angles.

Sample	1	2	3	4	5	6	7	8	9	10	11	12	13	14	15	16
A	16	16	4	16	16	16	4	4	4	16	16	16	4	4	4	4
B	14	14	14	14	14	4	4	4	14	14	4	4	14	4	14	4
C	60	30	30	30	60	30	60	60	30	30	60	60	60	30	60	30
D	300	300	280	280	280	280	280	300	300	300	300	280	280	300	300	280
E	2600	2600	1000	2600	1000	1000	1000	2600	2600	1000	1000	2600	2600	1000	1000	2600
angle	39.8	34.3	35.4	40.1	51.2	35.7	60.6	25.2	29.9	46.0	34.6	36.9	39.6	33.1	30.1	29.2

**Table 3:** Interpretation of the 2-level fractional factorial design using the contrast method.

Factor	Mean Value Factor Level 1	Mean Value Factor Level 1	Contrast
plasma nozzle speed in m/min	31.6	39.8	8.2
plasma nozzle distance in mm	39.0	32.5	6.5
volumetric flow in L/min	36.0	35.5	0.5
voltage in V	34.1	37.3	−3.2
rotational speed in U/min	37.1	34.4	−2.7
			**effect**
plasma nozzle speed × distance	34.6	36.9	2.3
plasma nozzle speed × volumetric flow	35.2	36.3	1.0
plasma nozzle speed × voltage	35.3	36.2	0.9
plasma nozzle speed × rotational speed	36.4	35.0	−1.4
distance × volumetric flow	35.5	36.0	0.5
distance × voltage	36.7	34.8	−1.9
distance × rotational speed	36.0	35.4	−0.6
volumetric flow × voltage	37.7	33.8	−3.9
volumetric flow × rotational speed	35.0	36.5	1.5
voltage × rotational speed	36.2	35.3	−0.9

**Table 4:** Printing parameters for the test structure.

Parameter	Value	Unit
diameter FLM nozzle	0.4	mm
FLM nozzle temperature	260	°C
layer thickness	0.2	mm
thickness 1st layer	0.25	mm
number of wall lines	4	-
number upper layers	1	-
number lower layers	2	-
filling	100	%
filling pattern	lines	-
filling speed	40	mm/s
wall speed	35	mm/s
speed 1st layer	12	mm/s
fan speed	25	%

**Table 5:** Plasma treatment (PT) parameter combination for sample production.

Parameter	PT1	PT2	PT3	PT4
plasma nozzle speed in m/min	12	4	4	1
plasma nozzle distance in mm	14	9	4	4
volumetric flow in L/min	45	45	60	60
voltage in V	280	290	300	300
rotational speed in U/min	2600	1800	2600	2600
contact angle	53.9°	28.6°	25.2°	24.3°

## Data Availability

Data are contained within the article.
